# Cardiac Cephalgia

**DOI:** 10.14740/cr361w

**Published:** 2014-12-04

**Authors:** Nancy Wassef, Ali Turab Ali, Alexia-Zacharoula Katsanevaki, Salman Nishtar

**Affiliations:** a Birmingham Heartlands Hospital, UK; b Kettering General Hospital, UK

**Keywords:** Coronary artery disease, Atherosclerosis and acute coronary syndrome

## Abstract

Although most of the patients presenting with ischemic heart disease have chest pains, there are other rare presenting symptoms like cardiac cephalgia. In this report, we present a case of acute coronary syndrome with an only presentation of exertional headache. It was postulated as acute presentation of coronary artery disease, due to previous history of similar presentation associated with some chest pains with previous left coronary artery stenting. We present an unusual case with cardiac cephalgia in a young patient under the age of 50 which was not reported at that age before. There are four suggested mechanisms for this cardiac presentation.

## Introduction

The term cardiac cephalgia (CC) or cephalalgia is a form of exertional headache precipitated by ischemic heart disease. Headache can be the only presentation of coronary artery disease (CAD). There are a total of 36 cases that have been reported in literature so far. The diagnosis depends on the presence of severe headaches worsened by physical exercise or stress, and relieved with rest and/or nitrate administration.

## Case Report

**Figure 1:**
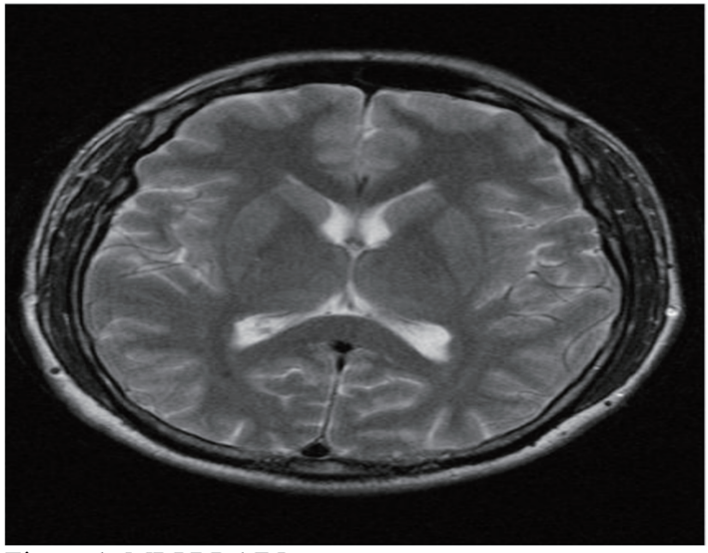
MRI of brain.

**Figure 2:**
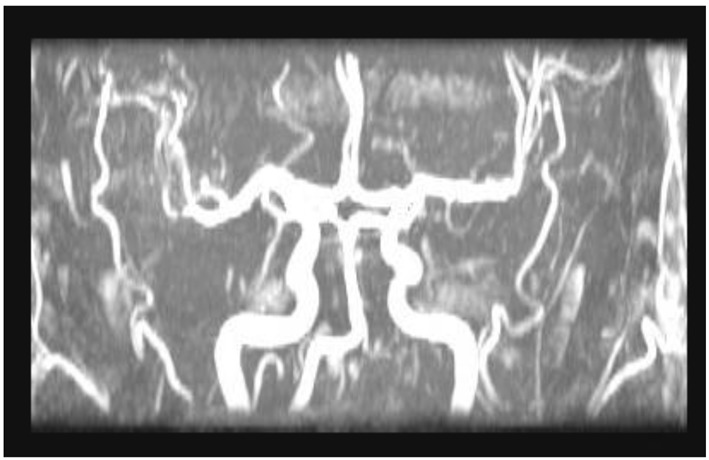
MRA of brain

A 44-year-old male patient presented to the cardiology outpatient clinic with exertional severe pressure headaches and mild chest discomfort relieved after resting. The symptoms were triggered only by strenuous exertion like brisk walking or playing table tennis. He was a non-smoker and there was no past medical or cardiac history of note apart from family history of diabetes mellitus. However, he was noted to have hyperlipidemia on subsequent blood tests. Neurological and cardiological examinations were also unremarkable. He was investigated for the headaches by MRI and MRA of brain, both of which showed no abnormalities (Fig. 1, 2). His random total cholesterol was elevated at 6.6 mmol/L and his electrocardiogram revealed normal sinus rhythm with no acute ischemic changes or evidence of previous infarction.

**Figure 3:**
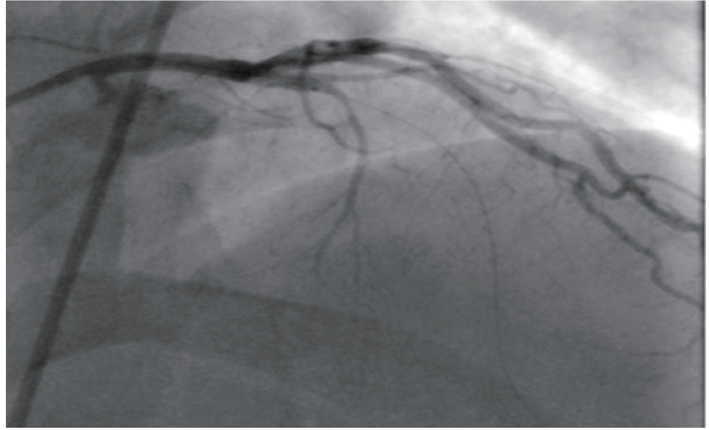
Totally occluded LAD.

**Figure 4:**
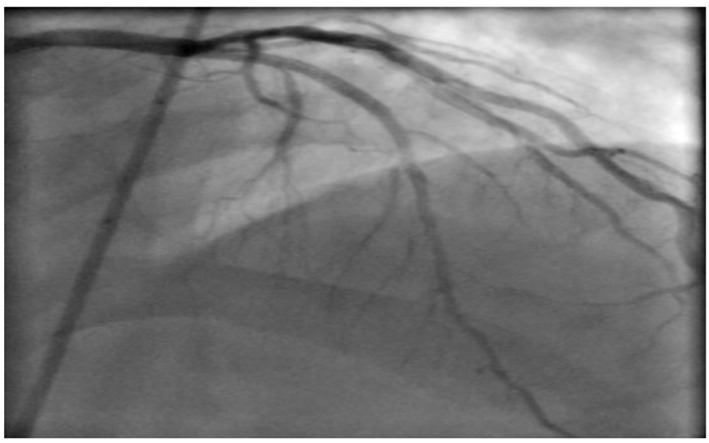
Post LAD stenting.

He was further investigated for suspected CAD with an exercise tolerance test, which showed non-diagnostic lateral ST segment depression associated with pressure headaches and chest discomfort, which resolved in few minutes during recovery. We therefore proceeded with coronary angiography which showed chronic total occlusion in the proximal left anterior descending (LAD) with normal left circumflex (LCX) and right coronary artery (RCA) (Fig. 3). The LAD was successfully treated with a single drug eluting stent with thrombolysis in myocardial infarction (TIMI) 3 flow (Fig. 4).

The patient was discharged on dual anti-platelet therapy and secondary preventive medications in addition to life style advice. At 4 months follow-up, the patient remained asymptomatic and resumed work and usual physical activities. Follow-up post procedure exercise tolerance test was reassuringly negative at a high workload.

**Figure 5:**
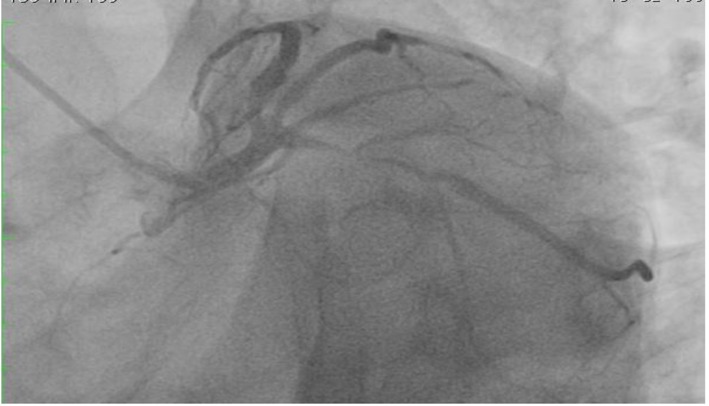
Severe proximal LCX lesion.

**Figure 6:**
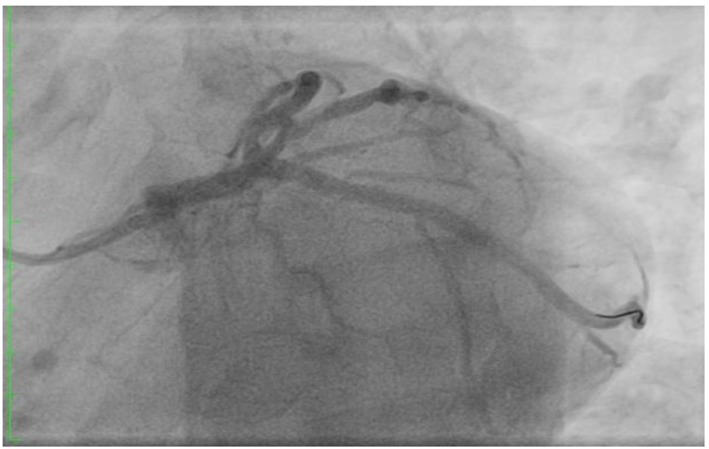
Post LCX stenting.

Two years later, he represented acutely with intermittent frontal pressure-like headaches on exertion relieved by rest without any associated chest pains or dypnea. His medications included aspirin, statin and a beta-blocker. There were no acute ischemic ECG changes and but given his previous history of CAD and CC, a 12-h cardiac troponin T was checked and it was raised at 0.026 μg/L (normal value, < 0.01). An inpatient coronary angiogram revealed patent LAD stent with TIMI 3 flow, but it showed a new severe lesion in the proximal LCX coronary artery (Fig. 5). This was successfully stented with a single drug eluting stent (Fig. 6).

### Differential diagnosis

It is important to distinguish CC from migraine with or without autonomic symptoms, and other forms of exertional headaches, cluster headache and thunderclap headache.

Subsequent use of triptans or ergot derivatives could be disastrous in patients presenting with CC.

## Discussion

The term CC or cephalalgia was coined by Lipton et al [1] in 1997 as a form of exertional headache and in total 36 cases have been reported in literature so far. The diagnosis of CC depends on the presence of severe headaches worsened by physical exercise or stress, and relieved with rest and/or nitrate administration. The International Headache Society has included CC as a specific entity in its international classification of headache disorders and proposed diagnostic criteria [2]: 1) headache, which may be severe, aggravated by exertion and accompanied by nausea and fulfilling criteria 3) and 4), 2) acute myocardial ischemia has occurred, 3) headache develops concomitantly with acute myocardial ischemia, and 4) headache resolves and does not recur after effective medical therapy for acute myocardial ischemia or coronary revascularization [2].

Bini et al’s [3] review of 30 cases with CC showed a mean age of 62.4 (range 35 - 85 years). Pain is usually not localized, and may be unilateral or bilateral. Pain is almost always severe and has been described as having different characteristics. There may or may not be other accompanying symptoms and if there are, 30% may be autonomic in nature. In 27% of the cases headache is the only manifestation of a cardiovascular ischemia. The headache starts immediately after physical exertion or on stress, and disappears gradually after resting. In 33% of cases headache appeared at rest. The frequency of this headache is highly variable and is concomitant with the acute cardiovascular event. Fifty-seven percent of patients show ECG abnormalities at rest [4] as well as elevated cardiac enzymes [5], and in the remaining ECG changes appear only during stress [6].

It is not surprising CC does not respond to simple painkillers [7], but promptly responds to nitrates. Triptans and ergot derivatives are contraindicated. In doubtful cases, the only test that can confirm the diagnosis is coronary angiography. If revascularisation is done CC may occur again in the event of coronary artery re-stenosis [8], or if there is another stenosis in another artery as was in our case.

Four theories have been proposed as a patho-physiologic mechanism. The first [1, 9] suggests that CC is a referred pain, as there is a connection between the central cardiac pathway (vagus nerve) and the cranial pain afferents (trigeminal nerve) in the upper part of the spinal cord. The second theory [1] proposes that CC is secondary to elevated intracranial pressure due to venous stasis caused by transient decrease in cardiac output due to ischemic ventricular dysfunction. According to third theory [1], it may be secondary to the local release in the heart muscle of chemical mediators capable of inducing remote pain, in this case headache. Among others, serotonin, bradykinin, histamine and substance P have been proposed as potential pain producing substances. The increase in intra-cardiac pressure during angina attacks could also result in release of natriuretic peptides with consequent vasodilatation of the cerebral vasculature resulting in headache. Finally CC could be due to the concomitant presence of vasospasm in both coronary and cerebral vascular beds [10]. When the headache occurs as the only manifestation of an acute coronary event, the diagnosis could be difficult; useful clues are older age at onset, no previous history of headaches, and presence of risk factors for cardiovascular disease and the onset of headache under stress. Knowledge of CC is scarce and it is very under recognized and under reported.

Differential diagnosis is from migraine with or without autonomic symptoms, tension type headache, primary and secondary forms of exertional headache, thunderclap headache and exacerbation of headache or migraine attacks by the use of nitroglycerine. It is extremely important to differentiate CC from other non-cardiac diseases, as the use of triptans or ergot derivatives could be serious. Our case has unique set of clinical characteristics; as unlike other cases the patient was younger than most of the cases described to date; presented twice (initially with worsening of stable angina and the second time with an acute coronary syndrome); predominantly with headache on the first occasion and solely with headache on the subsequent admission; both times he had objective evidence of ischemia and successfully treated on both occasions by percutaneous intervention requiring coronary stents, after which there was no recurrence of headache.

### Learning points

1) Headache could be an uncommon, but important cause of coronary ischemia. It may be the only manifestation without associated chest pains; response to nitrates aids diagnosis.

2) Useful clues are older age, no previous history of headache, presence of CAD risk factors, and symptoms on exercise or stress.

3) Distinguishing CC from migraine is important, as triptans and ergot derivatives are contraindicated.

4) Awareness of this condition is scarce as it is under recognized and under reported.
